# ART in Latin America: the Latin American Registry, 2020

**DOI:** 10.5935/1518-0557.20230025

**Published:** 2023

**Authors:** Fernando Zegers-Hochschild, Javier A Crosby, Carolina Musri, Fanny Petermann-Rocha, Maria do Carmo Borges de Souza, A Gustavo Martinez, Ricardo Azambuja, Armando Roque, Gustavo Estofan, Mario Vega Croker, on behalf of the Latin American Network of Assisted Reproduction

**Affiliations:** 1 Unit of Reproductive Medicine, Clínica Las Condes, Santiago, Chile; 2 Program of Ethics and Public Policies in Human Reproduction, Facultad de Medicina, Universidad Diego Portales, Santiago, Chile; 3 Centro de Investigación Biomédica, Facultad de Medicina, Universidad Diego Portales, Santiago, Chile; 4 Fertipraxis, Centro de Reproducao Humana, Rio de Janeiro, Brazil; 5 Medicina Reproductiva Fertilis, San Isidro, Buenos Aires, Argentina; Universidad de Belgrano, Buenos Aires, Argentina; 6 Fertilitat, Centro de Medicina Reprodutiva, Porto Alegre, RS 90620-130, Brazil; 7 Centro Especializado de Atención a la Mujer (CEPAM), Hacienda de las Palmas, Huixquilucan, Estado de México, Mexico; 8 CIGOR, Córdoba, Argentina; 9 Panama Fertility, Consultorios Hospital Punta Pacífica, Ciudad de Panamá, Panama; 10 Latin American Network of Assisted Reproduction (REDLARA), Montevideo, Uruguay

**Keywords:** ART registry, endometriosis, frozen embryo transfer, oocyte donation, perinatal mortality, preimplantation genetic testing

## Abstract

**Research question:**

What was the utilization, effectiveness and safety of assisted reproductive technology (ART) in Latin America during 2020?

**Design:**

Retrospective collection of multinational data on ART performed by 188 institutions in 16 countries.

**Results:**

Overall, 87,732 initiated cycles resulted in 12,778 deliveries and 14,582 births. The major contributors were Brazil (46.0%), Mexico (17.0%) and Argentina (16.8%). However, the highest utilization (cycles/million inhabitants) was Uruguay with 558, followed by Argentina with 490 and Panama with 425 cycles/million. Globally, women aged ≥40 years increased to 34% while women ≤34 dropped to 24.7%. After removing freeze-all cycles, the delivery rate per oocyte retrieval was 14.8% for intracytoplasmic sperm injection and 15.6% for IVF. Single-embryo transfer (SET) represented 38.3% of all fresh transfers, with delivery rate per transfer of 20.0%; this increased to 32.4% for elective SET (eSET) and 34.2% for blastocyst eSET, compared with blastocyst elective double embryo transfer (eDET) of 37.9%. However, multiple births increased from 1% in eSET to 30.5% in eDET. Perinatal mortality increased from 7.7‰ in singletons to 24.4‰ in twins and 64.0‰ in triplets. Frozen embryo transfer (FET) represented 66.6% of all transfers, with a delivery rate/transfer of 29.0%, significantly higher than 23.9% after fresh transfers at all ages (*p*<0.0001). Preimplantation genetic testing, reported in 8920 cycles, significantly improved delivery rate and decreased miscarriage rates at all ages (*p*≤0.041), including oocyte donation (*p*=0.002). Endometriosis was diagnosed in 28.3% of cases. The delivery rate in 5779 women after removal of peritoneal endometriosis was significantly better than tubal and endocrine factors in women aged 35-39 (*p*=0.0004) and women aged ≥40 (*p*=0.0353).

**Conclusions:**

Systematic collection and analysis of big data in a south-south cooperation model allow regional growth by implementing evidence-based reproductive decisions.

## INTRODUCTION

This is the 32^nd^ report of the Latin American Registry of Assisted Reproduction (RLA), which started in 1990 as the first multinational and regional registry of assisted reproductive technology (ART). Since 2012, reports have been published simultaneously in *Reproductive BioMedicine Online* and *JBRA Assisted Reproduction*, the official journal of the Latin American Network of Assisted Reproduction (REDLARA). As in previous years, this report provides information on the utilization, availability, effectiveness, safety and perinatal outcomes of ART treatments initiated between 1 January and 31 December 2020, and babies born up to September 2021. This report provides some additional information on the relationship between endometriosis in its different forms and the clinical outcome of ART procedures.

ART = assisted reproductive technology; FET = autologous frozen embryo transfer; FP = fertility preservation; FRESH = initiated fresh autologous IVF/ICSI cycles; FTO = embryo transfer cycles with autologous and donated vitrified/warmed oocytes; ICSI = intracytoplasmic sperm injection; OD = oocyte donation with fresh or frozen/thawed embryos; RLA = Latin American Registry of Assisted Reproduction.

## MATERIALS AND METHODS

Data on ART were collected from 188 centres in 16 countries in Latin America (Supplementary Table 1), covering fresh autologous cycles of IVF and intracytoplasmic sperm injection (ICSI); preimplantation genetic testing (PGT); frozen embryo transfer (FET) preceded by both fresh embryo transfer cycles and from freeze-all cycles; oocyte donation, including the transfer of fresh and frozen/thawed embryos; fertility preservation; and vitrified/warmed oocyte cycles (FTO), both autologous and heterologous.

All institutions reporting to RLA have been accredited by an independent body within REDLARA. The forms used for this process can be accessed on www.redlara.com Participating centres agree to have their data published by RLA and so no specific consent forms were requested for the scientific disclosure of data. The method of data collection in 2020 resembles that of previous years (Zegers-Hochschild *et al*., 2020), making results comparable. The definitions used are those published in the International Glossary on Infertility and Fertility Care (Zegers-Hochschild *et al*., 2017). When calculating clinical pregnancy or delivery rates per oocyte retrieval, cases resulting in total embryo freezing were not included in the calculation.

In order to study the relationship between endometriosis and ART outcomes, modifications were introduced in the data collection system. This is the first year in which more detailed information on the type of endometriosis was registered, including additional information on how the diagnosis was reached (clinical/ultrasound or surgical), as well as its type and localization (peritoneal, ovarian, deep infiltration) and the type of surgery performed.

The cumulative delivery rate was calculated from aspirations and their related fresh and frozen transfer cycles taking place between January and December 2020. We considered the first delivery after the transfer of either fresh or frozen/thawed embryos, or both, obtained after a reference oocyte retrieval. Only centres providing a permanent identification number were included in this calculation. In this year, cumulative deliveries were calculated from longitudinal data provided by 141 institutions in 15 countries. Results are expressed as: (i) cumulative delivery rate starting with all fresh transfers; and (ii) cumulative deliveries including only women having surplus frozen embryos apart from their fresh transfers.

Utilization of ART is expressed as the total number of cycles performed per million inhabitants. Considering that not all cycles carried out in every country were reported to the RLA, the best possible estimate of the non-reported cycles was obtained through information provided by regional directors of REDLARA, embryologists, clinicians and industry representatives. The magnitude of the estimates, which constitutes a potential source of error, is expressed as degrees of confidence according to Dyer *et al*. (2019) and later applied by Zegers-Hochschild *et al*. (2021).

For the purpose of visualizing the influence of women’s age on delivery rate, a general equation of the straight line was used to calculate the slope of decrease in delivery rate as age increases.

To test for the effect of age, number of embryos transferred and stage of embryo development at transfer on the delivery rate per embryo transfer, Poisson regression models with robust SE were used when analysing cross-sectional associations. The results are reported as prevalence ratios with their 95% confidence intervals (CI). Poisson regression models with robust SE were used because they provide prevalence ratio estimates that are relatively easy to interpret, rather than odds ratios (Grant, 2014). Robust SE were used to correct underinflation when applying the Poisson model for binary outcomes. When variables were not stratified by age, analyses were adjusted for it. *p*<0.05 was considered statistically significant and STATA 17 (StataCorp LP, College Station, TX, USA) was used to perform all analyses.

## RESULTS

A total of 188 centres in 16 countries reported 87,732 initiated cycles during 2020, resulting in 12,778 deliveries and 14,405 live births. This represents one more country than in previous years, following the incorporation of Costa Rica. Overall, there was a drop of eight centres and 19,188 ART cycles, resulting in 8441 fewer babies born. This is largely the result of the transitory and/or definitive closure of centres associated with the COVID-19 pandemic. In fact, this is the first time there has been a drop in the number of cycles and centres reporting. Regional trends remain unchanged, and Brazil is still the largest contributor with 46.0% of all initiated cycles, followed by Mexico and Argentina with 17.0% and 16.8% of cycles, respectively (Table 1). Fresh-initiated IVF and ICSI cycles still predominate with 45% of initiated cycles, followed by 25.8% of FET and 15.3% of oocyte donation. As will be seen later in this manuscript, this relatively high proportion of cycles, including reproductive donation, is related to a high proportion of women ≥40 (34%), compared with only 18% in Europe in 2018 (European IVF Monitoring Consortium, 2022) and approximately 26% in the USA, as reported by SART in 2022 (https://www.sartcorsonline.com/rptCSR_PublicMultYear.aspx?reportingYear=2020).

**Table 1:** Treatment with art reported in Latin America, 2020.

Country	Centres	FP	FRESH	FET	OD	FTO	Total	Deliveries registered by RLA	Estimated total number of deliveries from ART	Estimated proportion of births from ART/total births in the country
Argentina	22	1279	6080	3275	3675	471	14,780	1995	2563	0.48
Bolivia	3	6	216	24	161	21	428	82	172	0.08
Brazil	67	4813	19,520	11,965	2572	1484	40,354	5054	5274	0.20
Chile	11	565	2079	1370	600	273	4887	829	1066	0.55
Colombia	14	167	1121	629	524	91	2532	464	592	0.09
Costa Rica	1	6	36	5	3	1	51	5	29	0.05
Ecuador	4	105	322	81	62	57	627	107	171	0.06
Guatemala	2	13	137	101	75	1	327	84	148	0.04
Mexico	41	640	6656	3337	3923	316	14,872	2679	3580	0.22
Nicaragua	1	13	52	26	8	8	107	21	27	0.01
Panama	3	78	409	209	135	31	862	145	252	0.36
Paraguay	1	69	164	124	49	14	420	44	85	0.13
Peru	13	1091	2007	1032	1256	548	5934	947	1357	0.32
Rep. Dominicana	2	9	91	33	56	2	191	33	39	0.03
Uruguay	2	61	514	422	277	55	1329	278	383	0.79
Venezuela	1	0	14	10	7	0	31	11	177	0.03
Total (%)	188	8915 (10.2)	39,418 (44.9)	22,643 (25.8)	13,383 (15.3)	3373 (3.8)	87,732	12,778	15,915	

**Figure 1:**
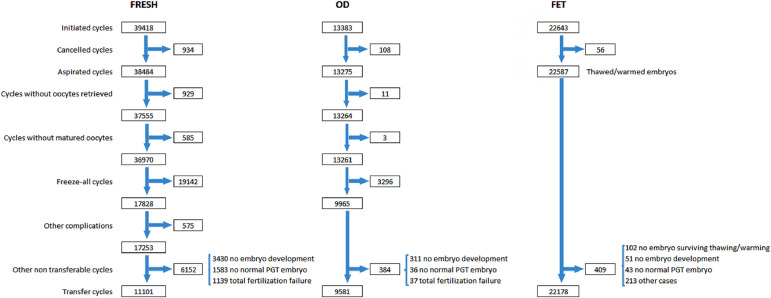
Events that affect the outcome of fresh IVF and ICSI (IVF/ICSI), fresh and frozen oocyte donation and autologous frozen embryo transfer in Latin America, 2020. FET=frozen embryo transfer; FRESH=initiated fresh autologous IVF/ICSI cycles; OD=oocyte donation; PGT=preimplantation genetic testing (PGT-A, PGT-M, PGT-SR reported together).

Given that not all initiated cycles are intended to result in an immediate pregnancy, and not all oocytes collected can be fertilized or the resulting embryos transferred, pregnancy rate and delivery rate are directly affected by how selective the denominator is. In order to understand and interpret the outcome under different treatment modalities, Figure 1 provides the sequence of events that need to be considered when looking at the outcome with a specific technique (IVF/ICSI, oocyte donation, FET), starting with: initiated cycle; cancellations before follicle aspiration; aspirations with or without mature oocytes; freeze-all oocytes, embryos, or both; the number of cycles with fertilized oocytes or failed fertilization; and the number of cycles with viable embryos for transfer or normal embryos after PGT. After all these events have been considered and adjusted for, pregnancy and delivery rates can be calculated with a well-established denominator: initiated, aspirated and transfer cycles. This detailed description, however, is only possible in a cycle-based data collection system.

### Use of ART in Latin America

**Figure 2:**
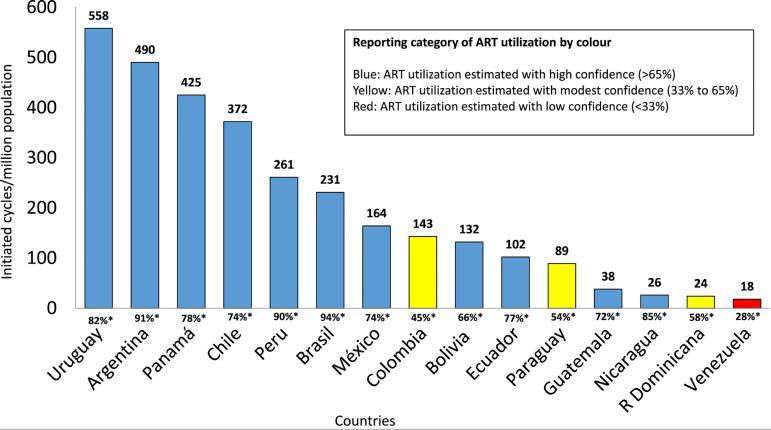
Use of assisted reproductive technology (ART). Estimated number of initiated cycles per million inhabitants by country in Latin America, 2020. *Rate of reporting = number of cycles reported to the registry / total or estimated total number of cycles performed in the country.

As seen in Figure 2, the RLA collects data on a vast proportion of ART cycles carried out in most countries in the region; in particular, it covers between 74% and 94% of the major contributors. Overall, Uruguay and Argentina, two countries with laws providing universal care to ART, have the highest utilization, with 558 and 490 cycles per million inhabitants, respectively, followed by Panama, with 425 cycles/million inhabitants. Brazil is by far the major contributor in the region, but its utilization is still very poor (231 cycles/million population).

### Age of women treated in Latin America

**Figure 3:**
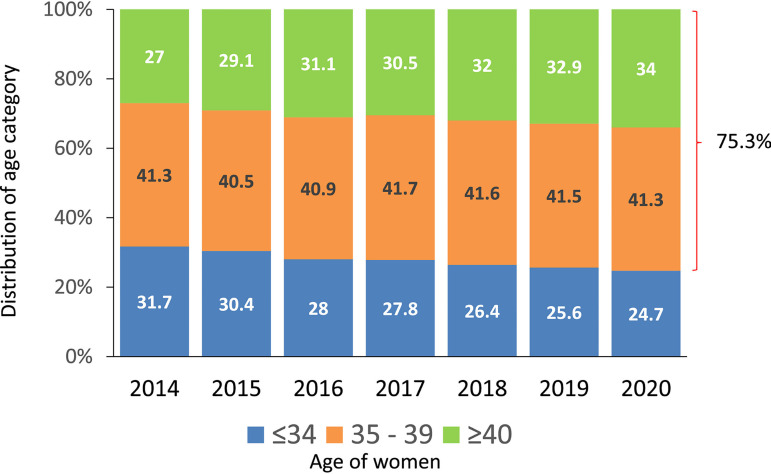
Age distribution of female partner in fresh IVF and intracytoplasmic sperm injection (IVF/ICSI) in Latin America, 2014-2020.

As seen in Figure 3, in the last 7 years, the proportion of women ≤34 has dropped from 31.7% to 24.7%; women ≥40 have continued to increase, from 27% to 34%. According to this, 75.3% of women treated in the region were 35 years or older, with profound variations among countries. The proportion of women ≥40 in the major contributors were Brazil 35.3%, Mexico 25.3%, Argentina 41.9% and Peru 40.4% (data not shown here). This is very important when comparing treatment outcomes in different countries and regions. The proportion of women ≥40 is only 18% in Europe and approximately 26% in the USA (European IVF Monitoring Consortium, 2022, and https://www.sartcorsonline.com/rptCSR_PublicMultYear.aspx?reportingYear=2020, respectively).

### Outcome of autologous fresh IVF and ICSI cycles according to the age of women and number of embryos transferred

In 2020, there were 39,418 fresh-initiated IVF/ICSI cycles, but as reported in Figure 1, after discarding cancelled cycles, freeze-all cycles and other conditions impeding embryo transfer, the number of cycles where at least one mature oocyte was collected dropped to 17,253. Furthermore, after discarding cases with failed fertilization, no embryo development and PGT cases without normal embryos, the number of transfer cycles was further reduced to 11,101. Table 2 provides clinical pregnancy rates (CPR) and delivery rates per oocyte retrieval and embryo transfer according to the age of women and the type of fertilization process. Consistent with previous years, ICSI represents 84.8% of transfers. This high proportion of ICSI, without a clear explanation apart from the fear of fertilization failure, has had small changes over the last decade (85.7% in 2010; https://redlara.com/registro.asp). When stratified by the age of the female partner, the pregnancy rate by oocyte retrieval was significantly higher in IVF than in ICSI only in women ≥35 years (*p*<0.0001). However, there were no differences in the delivery rate by oocyte retrieval and delivery rate by embryo transfer. As expected, the chances of achieving a delivery decreased with age.

**Table 2:** CPR and delivery rate in fresh autologous IVF and ICSI cycles stratified according to the age of women in 2020.

	Age of women	Oocyte retrievals	CPR per oocyte retrieval	Delivery rate per oocyte retrieval	Embryo transfers	Delivery rate per transfer
ICSI	≤34	3393	1206 (35.5%)	911 (26.8%)	2616	911 (34.8%)
	35-39	6019	1412 (23.5%)	1018 (16.9%)	3980	1018 (25.6%)
	≥40	5714	536 (9.4%)	310 (5.4%)	2510	310 (12.4%)
IVF	≤34	635	230 (36.2%)	156 (24.6%)	510	156 (30.6%)
	35-39	1148	331 (28.8%)	207 (18.0%)	876	207 (23.6%)
	≥40	919	136 (14.8%)	59 (6.4%)	609	59 (9.7%)

CPR = clinical pregnancy rate; ICSI = intracytoplasmic sperm injection.

**Figure 4:**
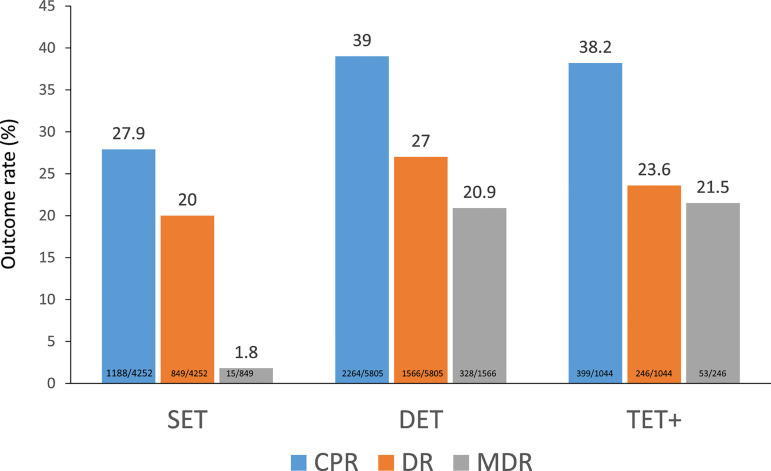
Clinical pregnancy rate (CPR), delivery rate (DR) and multiple delivery rate (MDR) per embryo transfer in IVF and ICSI cycles according to the number of embryos transferred in Latin America,2020. SET=single-embryo transfer; DET=double-embryo transfer; TET+=triple or more embryo transfer.

Of all fresh transfers, SET continued to increase, from 36.2% as reported in 2019 (Zegers-Hochschild *et al*., 2022), to 38.3% in 2020, and 90.6% of all fresh transfers included a maximum of two embryos (Figure 4). The effect of the number of embryos transferred on the CPR, delivery rate and multiple births can be seen in Figure 4. Both the CPR and delivery rate after DET were significantly higher than after SET (CPR: prevalence ratio 1.36; 95% CI 1.32-1.48; *p*<0.001) (delivery rate: prevalence ratio 1.35; 95% CI 1.26-1.45; *p*<0.001). However, its impact on multiple births increased from 1.8% of monozygotic twins (MZT) after SET to 20.9% of twins after DET and 21.5% after TET.

### Outcome of autologous IVF and ICSI after elective and non-elective SET and DET

There were 4252 SET, which were further stratified into eSET (when one embryo is chosen from a larger cohort of available embryos) and oSET (when one embryo is transferred because there are no more embryos available for transfer) and eDET over oDET (the transfer of only two embryos because there are no more embryos available for transfer). In this universe, eSET represented 39.5% of SET. As seen in Table 3, both CPR and delivery rates were significantly greater after eSET (42.8% and 32.4%, respectively) compared with oSET (18.2% and 11.9%, respectively) (*p* < 0.0001); and after eDET (50.3% and 35.8%, respectively) compared with oDET (30.9% and 20.7%) (*p* < 0.0001). These differences were accompanied by an almost three times higher rate of monozygotic twinning after oSET than eSET. Furthermore, when two embryos were transferred, the rate of twins was also significantly higher in eDET than oDET (*p* < 0.0001). The higher rate of dizygotic twins after eDET can be considered an indirect expression of higher embryo implantation rate associated with better embryo quality in women with the capacity to generate more embryos. When this comparison was made after the transfer of only blastocyst (Supplementary Table 2), the delivery rate after the transfer of eDET (37.9%) and eSET (34.2%) were only 3.7% points different. However, the rate of multiple births rose from 1% of MZT after blastocyst eSET to 30.5% after blastocyst eDET.

**Table 3:** CPR, delivery rate and gestational order in elective and non-elective SET and DET in fresh autologous IVF/ICSI in 2020.

Type of transfer	Embryo transfers		Clinical pregnancies		Deliveries							
	Number	%	Number	%	Number of deliveries	Delivery rate per embryo transfer (%)	Singleton (n)	Singleton (%)	Twin (n)	Twin (%)	≥Triplets (n)	≥Triplets (%)
oSET	2572	60.5	469	18.2	305	11.9	296	97.0	9	3.0	0	0
eSET	1680	39.5	719	42.8	544	32.4	538	98.9	6	1.1	0	0
oDET	3382	58.3	1046	30.9	699	20.7	599	85.7	96	13.7	4	0.6
eDET	2423	41.7	1218	50.3	867	35.8	639	73.7	225	26.0	3	0.3

CPR = clinical pregnancy rate; eDET = elective double-embryo transfers; eSET = elective single-embryo transfers; ICSI = intracytoplasmic sperm injection; oDET = the transfer of only two embryos because there are no more embryos available for transfer; oSET = transfer of only one embryo because there are no more embryos available for transfer.

**Figure 5:**
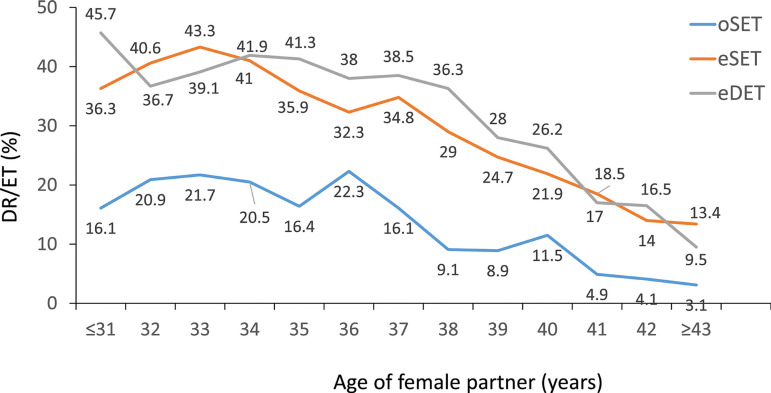
Delivery rate per embryo transfer (DR/ET) in IVF and ICSI cycles according to the age of the female partner and the number of embryos transferred in Latin America, 2020. eDET=elective double-embryo transfers; eSET=elective single-embryo transfers; ICSI=intracytoplasmic sperm injection; oSET=transfer of only one embryo because there are no more embryos available for transfer.

When examining the impact of the age of women, and consistent with the 2019 report, the delivery rate after transferring eSET was higher than after oSET at all ages (*p* = 0.0355 to *p* < 0.0001). Overall there was no significant difference in delivery rate of eDET compared with eSET (prevalence ratio 1.16; 95% CI 0.97-1.38; *p*=0.103). In women between 35 and 40 years, delivery rates of eDET were higher than eSET but the differences in this group were not statistically significant (Figure 5).

### Outcome of oocyte donation cycles

**Figure 6:**
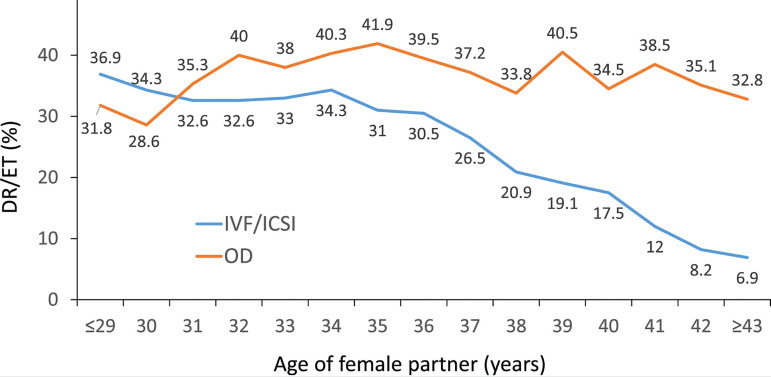
Delivery rate per embryo transfer (DR/ET) in fresh autologous IVF and intracytoplasmic sperm injection (ICSI) and fresh oocyte donation (OD) cycles according to the age of the female partner in Latin America, 2020.

As seen in Figure 1, there were 13,383 initiated cycles representing 15.3% of all cycles performed in the region. After discarding cancellations, freeze-all cycles and other factors, there were 9581 embryo transfers. In contrast with autologous reproduction, the delivery rate using donated oocytes was practically unaffected by the age of recipients (Figure 6). Furthermore, delivery rates and miscarriage rates were compared in oocyte recipients and in a selected population of women ≤34 years with autologous reproduction. To homogenize both populations, only FET cycles were used. In the absence of PGT, the miscarriage rate in oocyte recipients (18.2%) was significantly greater than in a subset of autologous reproduction in women ≤34 years (14.9%) (*p*=0.002). In the same way, the delivery rate by embryo transfer was significantly lower in oocyte recipients (29.3%) compared with women ≤34 years (32.7%) (*p*<0.001). Furthermore, in a subset of women where PGT was performed, there were no differences in miscarriage rate in oocyte recipients (11.9%) and women ≤34 years with their own eggs (11.1%). The delivery rates in these two groups (39.6% and 40.9%) were also not significantly different. Therefore, in this very young female population, the use of PGT significantly reduced the rate of miscarriage and increased delivery rates, both in autologous cycles and in oocyte recipients (Table 4). When comparing outcomes according to the number of embryos transferred, the CPR, delivery rate and multiple births in 3091 fresh transfers and 6490 frozen/thawed transfers can be seen in Supplementary Tables 3 and 4.

**Table 4:** Effect of PGT on the delivery rate and miscarriage rate according to age of women in autologous FET and OD FET (2020).

	Age of women	FET with PGT	FET without PGT	PR (95% CI); *p*-value
Miscarriage^a^	Oocyte donors	11.9% (54/452)	18.2% (435/2391)	1.53 (1.17, 1.90); 0.002^b^
	Autologous ≤34	11.1% (47/424)	14.9% (394/2640)	1.35 (1.01, 1.79); 0.041^b^
	Autologous 35-39	11.1% (98/879)	16.6% (516/3112)	1.49 (1.21, 1.82); <0.001^b^
	Autologous ≥40	13.9% (94/675)	21.9% (317/1449)	1.57 (1.27, 1.94); <0.001^b^
Delivery^a^	Oocyte donors	39.6% (352/890)	29.3% (1642/5600)	0.74 (0.68, 0.82); <0.001^c^
	Autologous ≤34	40.9% (329/805)	32.7% (1866/5707)	0.80 (0.73, 0.88); <0.001^c^
	Autologous 35-39	36.4% (679/1866)	27.6% (2132/7724)	0.76 (0.71, 0.81); <0.001^c^
	Autologous ≥40	35.6% (513/1440)	19.5% (904/4636)	0.55 (0.50, 0.60); <0.001^c^

FET = frozen embryo transfer; OD FET = oocyte donation frozen embryo transfer;

PGT = preimplantation genetic testing; PR = prevalence ratio.

a For miscarriage the denominator is clinical pregnancies; for deliveries, the denominator is embryo transfers.

b Likelihood of having a miscarriage. The reference group is ‘with PGT’.

c Likelihood of delivery. The reference group is ‘with PGT’.

**Figure 8:**
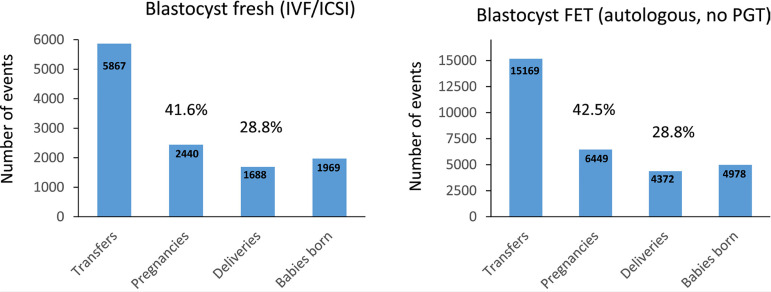
Clinical pregnancy rate, delivery rate and babies born after fresh and frozen-thawed blastocyst transfers in Latin America, 2020. FET=frozen embryo transfer; ICSI=intracytoplasmic sperm injection; PGT=preimplantation genetic testing.

The better outcome after FET was multifactorial, but in this case, it results from a much higher proportion of blastocyst transfers in FET (19,253/22,178; 86.8%) compared with fresh transfers (5917/11,101; 53.3%). This finding is reassuring because when comparing the outcome after blastocyst transfer in a fresh and FET cycle (without PGT), both CPR and delivery rates showed no significant difference (CPR: prevalence ratio 0.98; 95% CI 0.93-1.02; *p*=0.349; delivery rate: prevalence ratio 1.00; 95% CI 0.94-1.06; *p*=0.964) (Figure 8). It is thus likely that the better results seen in FET over fresh transfers was a consequence of a much higher proportion of blastocyst transfers in the former.

During 2020 there were 19,142 autologous freeze-all cycles (Figure 1), and a total of 7484 FET resulting from autologous freeze-all procedures performed in 2020 and in previous years. There were 2092 deliveries with an overall delivery rate per transfer of 28.0% (Supplementary Table 6). Furthermore, 810 women had more than one transfer from embryos originating from the same freeze-all procedure. The cumulative delivery rate in this subgroup reached 30.4% in spite of a mean age of 37.5 (5.39) years.

In order to compare the outcome of freeze-all cycles and FET cycles resulting from failed fresh transfers, all cases where PGT was performed were excluded from the calculation. There were 10,476 autologous FET transfers and 2772 deliveries, with a delivery rate of 26.5%, compared with a delivery rate of 28% in freeze-all cycles; this is significantly greater (*p*=0.0258), demonstrating that when the best embryos are selected for delayed transfer, the chances of delivery are even greater than after fresh transfers (Figure 4).

### Influence of blastocyst transfer cycles

**Figure 9:**
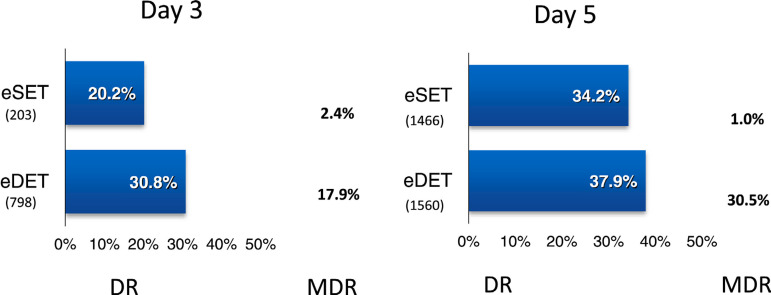
Delivery rate (DR) and multiple delivery rate (MDR) per embryo transfer in IVF and ICSI cycles according to eSET and eDET and the day of embryo transfer in Latin America, 2020. eDET=elective double-embryo transfer; eSET=elective single-embryo transfers.

The proportion of blastocyst transfers over cleaving embryos increases year after year. It represented 30.3% of all transfers in 2016, increasing to 77.6% in 2020; and as mentioned before, in cases of FET, it represents 86.8% of all transfers compared with 53.3% in fresh IVF/ICSI. In oocyte donation cycles (both fresh and frozen), the proportion of blastocyst transfers reached 74.7%. When comparing the delivery rate and multiple birth rate after the elective transfer of 8-cell cleaving embryos (day 3) and elective transfer of day 5 blastocysts in IVF and ICSI cycles, the delivery rates were significantly higher after the transfer of blastocysts, both in eSET and eDET (eSET: prevalence ratio 1.69; 95% CI 1.27-2.24; *p*<0.001; eDET: prevalence ratio 1.23; 95% CI 1.08-1.39; *p*<0.001) (Figure 9). Furthermore, following eDET the proportion of multiple births was also significantly higher after blastocyst transfer (30.5% compared with day 3 cleaving embryos [17.9%], *p*<0.001).

### Influence of PGT on ART outcome

**Figure 10:**
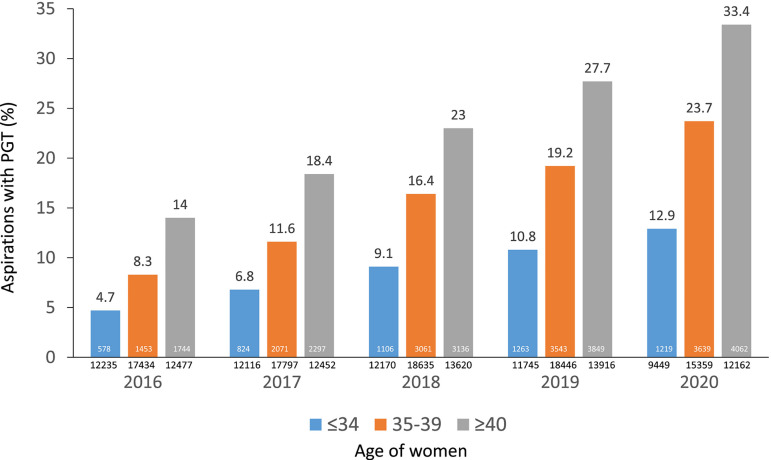
Five year trends in the use of preimplantation genetic testing (PGT) in autologous fresh cycles for aspirations with at least one mature oocyte in different age groups in Latin America, 2016-2020.

In the last 5 years, the proportion of aspirations leading to PGT has increased almost 2.5 times in all age categories (Figure 10). In 2020, a total of 144/188 centres (76.6%) reported 8920 aspirations of autologous fresh cycles where PGT was performed. This corresponds to 24.1% of aspirations with at least one mature oocyte. When stratified by age, the percentage of aspirations with PGT was 12.9% in women ≤34, 23.7% in women 35-39 years and 33.4% in women ≥40 years (Figure 10). Furthermore, there were 5094 embryo transfer cycles, of which 4178 transfers were from autologous cycles (82%) and 916 (18%) from oocyte donation. The mean age of women undergoing autologous PGT was 38.3 (SD 3.97); and the age distribution included 17.6% in women ≤34 years, 20.2% in women 35 to 37 years, 19.7% in women 38 and 39 years and 42.5% in women ≥40 years. In oocyte donation, the mean age of donors was 25.5 (SD 4.75).

Overall, there were 27,287 embryos examined. Out of 5114 embryos in women ≤34 years, the proportion of normal embryos was 50.2%. Out of 11,990 embryos in women 35-39 years, the proportion of normal embryos was 40.1%. In women ≥40 years, out of 10,183 embryos, the proportion of normal dropped to 22.9%. Furthermore, in 3166 embryos generated from oocyte donors, the proportion of normal embryos was 63.9%. The effect of PGT on the delivery rate and miscarriage rate can be seen in Table 4. When stratified by age, PGT significantly decreased miscarriage in all age categories, including women under 34 years (*p*=0.041), and oocyte donation (*p*=0.002). Concerning the effect of PGT on the probability of achieving birth, the differences in deliveries with and without PGT are again significantly greater with PGT at all age groups, including oocyte donation (*p*<0.001) (Table 4).

### Influence of endometriosis on the outcome of ART

Endometriosis was present, either as a primary or secondary diagnosis, in 11,153 out of 39,418 initiated fresh cycles (28.3%). Of these, peritoneal endometriosis diagnosed via laparoscopy comprised 11,040 (99%); there were 45 cases of partial oophorectomy and either aspiration or removal of endometriotic cysts. There were also 41 cases of surgery for deep infiltrating endometriosis and 24 cases of a combination of these categories. Given that severe endometriosis was reported in very few cases, a comparison was made between the outcome of cases where peritoneal endometriosis was managed by laparoscopic surgery and a ‘control group’ of tubal and endocrine factors excluding premature ovarian insufficiency (Supplementary Table 7). In this ‘control group’, cases with a secondary diagnosis of endometriosis were also ruled out. Similarly, cases included in peritoneal endometriosis did not have other associated diagnoses. Supplementary Table 7 provides information on the numbers and the mean number of oocytes collected, as well as the delivery rates in these two groups of women, stratified by age categories. Although the mean number of oocytes collected in women ≤34 and ≥40 years was significantly lower in the presence of endometriosis (≤34: 9.3 [6.274] *versus* 11.6 [7.201]: *p*<0.0001; 95% CI 2.1171-2.4829; ≥40: 5.2 [4.415] *versus* 6.0 [5.327]: *p*<0.0001; 95% CI 0.6363-0.9637), the delivery rate per embryo transfer was 38.3 *versus* 33.9 (*p*=0.0744; 95% CI -0.4378 to 9.1025) in the ≤34 years age group and it was significantly greater in women ≥34 years; 35-39: 31.2 *versus* 24.1: *p*=0.0004; 95% CI 3.2333 to 10.8003 and ≥40: 16.8 *versus* 12.2: *p*=0.0353; 95% CI 0.3185 to 8.3988.

### Cumulative delivery rate

**Figure 11:**
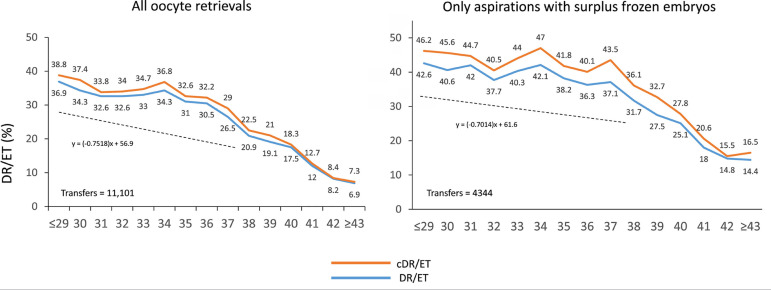
Cumulative delivery rate (cDR) per aspiration cycle and delivery rate per fresh embryo transfer (DR/ET) in IVF and ICSI cycles according to the age of the female partner in Latin America, 2020. (a) All aspirations irrespective of whether there were frozen embryos for further transfer. (b) Only aspirations with surplus frozen embryos. The equation represented by a dotted line is a reflection of the slope of decrease in delivery rate between women of 29 years and younger and women up to 38 years of age.

Cumulative delivery rates were calculated in the first cohort of 11,101 aspiration cycles irrespective of whether women had surplus frozen embryos for delayed transfer, and in a subgroup of 4344 women who, apart from their fresh transfers, had supernumerary embryos frozen for further transfers, irrespective of whether they were used during 2020. To calculate cumulative deliveries, this latter group is the one that better reflects what cumulative chances are, because women that do not have frozen embryos had their only chance after the fresh transfer. As seen in Figure 11, the delivery rate per fresh transfer is notably higher at all ages in women having surplus frozen embryos compared with all women, including a high proportion of aspirations without surplus embryos (60.9%). As expected, the delta between fresh and cumulative outcome was further increased in the selected cohort of women having frozen embryos for delayed transfer. Another interesting observation in this subcohort of women having fresh and frozen embryos was the less pronounced slope of the drop in deliveries as age increases. As seen in Figure 11, the effect of age on the chances of delivery was less prominent in women who generated more embryos.

**Figure 12:**
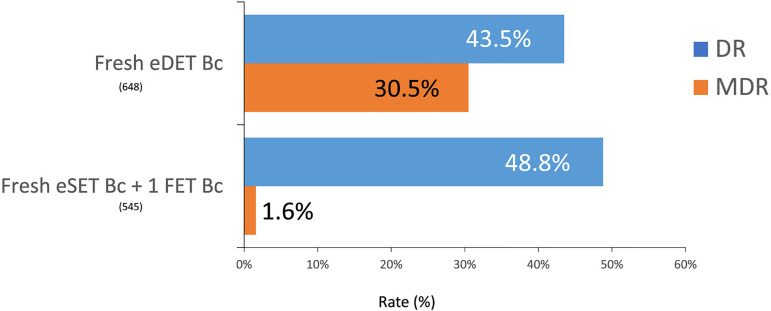
Delivery rate (DR) and multiple delivery rate (MDR) after the transfer of two fresh elective blastocysts (eDET Bc) or one fresh elective blastocyst + 1FET blastocyst (eSET Bc+1FET Bc) in women under 35 years of age in Latin America, 2020.

Cumulative delivery rates reached 48.8% in a subset of 545 women ≤34 years with only one fresh (eSET) and one frozen/thawed blastocyst transferred; compared with 43.5% when two fresh blastocysts were simultaneously transferred in 648 women. Furthermore, multiple births increased from 1.6% of MZT in cumulative blastocyst SET to 30.5% after a fresh blastocyst DET (Figure 12).

### Perinatal outcome and preterm birth

**Figure 13:**
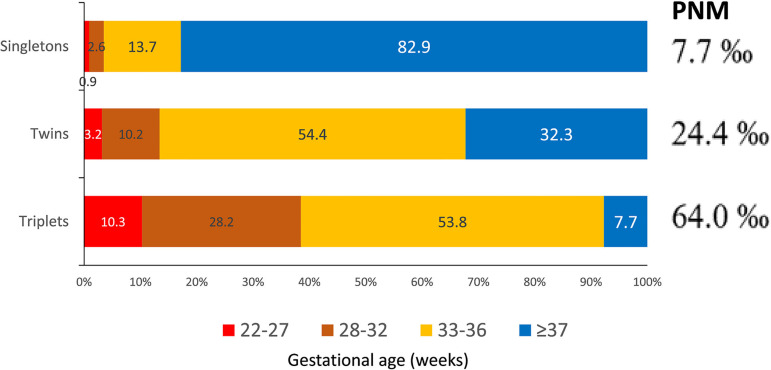
Preterm birth and perinatal mortality (PNM) according to order of gestation and gestational age in Latin America, 2020.

Perinatal mortality (PNM) was calculated from 12,778 deliveries and 14,582 births. Of these, 75.6% of newborns were singletons; 23.6% were twins and 0.9% triplets or more. PNM is consistent with previous years, with 7.7‰ of perinatal deaths in singletons, rising to 24.4‰ in twins and 64.0‰ in triplets and more (Table 5). On the other hand, preterm birth (Figure 13) took place in 17.2% of singletons, rising to 67.8% in twins and 92.3% in triplets. Of these, extreme preterm births (≤33 weeks of gestation) increased from 3.5% in singletons to 13.4% and 38.5% in twins and triplets, respectively. The negative impact on the health of mothers and children born from preterm and extreme preterm births has been described in detail by Sazonova *et al.* (2013) and the Practice Committee of the Society for Reproductive Endocrinology and Infertility, Quality Assurance Committee of the Society for Assisted Reproductive Technology, and the Practice Committee of the American Society for Reproductive Medicine (2022).

**Table 5:** Perinatal mortality according to gestational order in 2020.

Outcome	Singleton	Twin	≥ Triplet
Live birth^a^	10,932	3356	117
Stillbirth	31	27	4
Early neonatal death	54	57	4
Perinatal mortality^b^	7.7‰	24.4‰	64.0‰

a Early neonatal deaths are excluded.

b Perinatal mortality = (stillbirth + early neonatal death) / (live birth + stillbirth + early neonatal death).

### Influence of FET cycles

**Figure 7:**
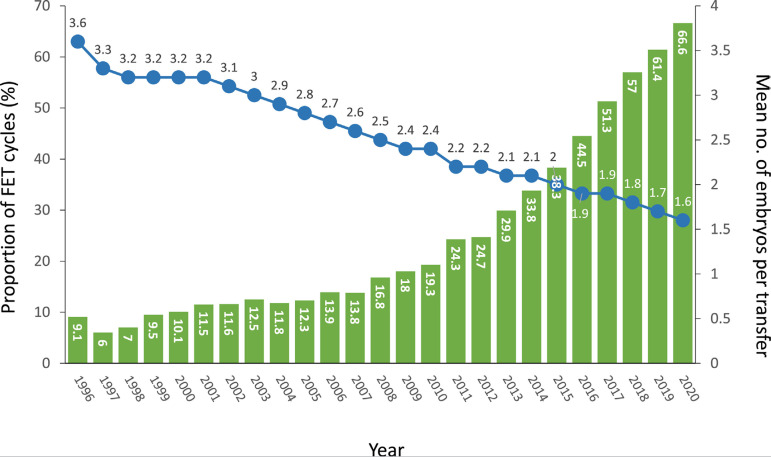
Proportion of frozen embryo transfer (FET) cycles and the mean number of embryos transferred in fresh cycles in Latin America, 1996-2020.

In 2020, there were 22,643 initiated FET cycles, representing 25.8% of all procedures (Table 1) and 66.6% of all autologous transfers (Figure 1). This represents a consistent increment over the past 25 years (Figure 7). In this same time interval, the mean number of embryos transferred in fresh cycles dropped from 3.6 in 1996 to 1.6 in 2020 (Figure 7). Of all initiated FET cycles, 465 (2.1%) were discontinued. Reasons for discontinuation are described in Figure 1. Therefore, out of 22,178 FET cycles, the overall CPR and delivery rate per transfer were 41.4% and 29.0%, respectively (Supplementary Table 5). The higher CPR and delivery rate in FET compared with fresh transfers are observed across all numbers of embryos transferred (Figure 4 and Supplementary Table 5). This better outcome in FET over fresh transfers (delivery rate/transfer 29.0% and 23.9%, respectively) is significantly higher at all ages (*p*<0.001). This is also accompanied by a reduction in multiple births. Out of 6423 FET deliveries reported in this period, 88.1% were singletons, 11.7% were twins and 0.2% were triplets and higher (Supplementary Table 5), compared with 85.1% of singletons, 14.5% twins and 0.4% triplets and higher after 2661 deliveries in fresh autologous transfers (data not shown here). Differences between singletons and between twins are highly significant (*p*=0.0001 and *p*=0.0002, respectively).

### Fertility preservation

**Figure 14:**
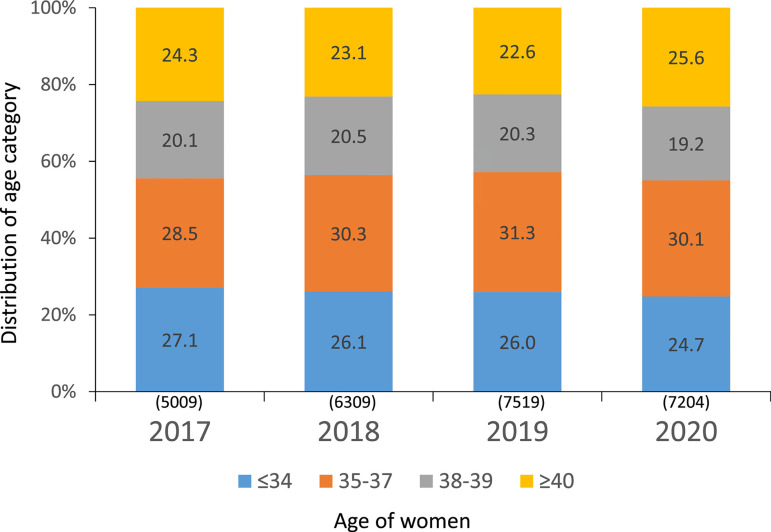
Fertility preservation cycles per year according to the age of women in Latin America, 2017-2020. Numbers include only cycles where at least one mature oocyte was collected.

A total of 7558 initiated cycles of oocyte vitrification for fertility preservation were reported, of which 7204 had at least one mature oocyte (95.3%). The age distribution of women has shown minimal changes over recent years and the proportion of women trying to preserve their fertility at ≥38 years remains very high (44.8%) (Figure 14). As expected, the mean number of vitrified oocytes decreased with age. The mean (SD) numbers of metaphase II vitrified oocytes was 7.04 (5.83), with ample variations according to women’s age. In women ≤34, the mean was 9.02 (7.05); in women 35-38 was 7.32 (5.73); 39-40 years was 5.77 (4.52) and in women ≥40 was 4.54 (3.76) oocytes. In 95.1% of cases, the reason for oocyte vitrification was a postponement of fertility for reasons other than cancer, which represented the primary reason for fertility preservation in 4.9% of cases (data not shown here).

## DISCUSSION

This is the 32^nd^ report on ART procedures performed in Latin America. As a result of the COVID-19 pandemic, the number of new centres reporting to RLA as well as the total number of cycles dropped for the first time in three decades. Some centres restricted medically assisted reproduction to non-ART procedures, while others definitely closed. Other centres had to restrict their personnel, making reporting more difficult. During this reporting year, one centre from Costa Rica has been incorporated in REDLARA, after ART was re-established in that country following the ruling by the Inter-American Court of Human Rights in favour of IVF (http://www.corteidh.or.cr/docs/casos/articulos/seriec_257_esp.pdf).

The best estimate for ART utilization by country is depicted in Figure 2. Uruguay and Argentina continue to have the highest utilization due to laws providing free access. However, in spite of this, economic restrictions in low or middle income (LMIC) countries limit access to ART for a wider population. The mean number of ART cycles per million in 15 Latin American countries (204 cycles/million) is only 14.6% of the mean utilization of 1400 cycles per million in 21 European countries with full reporting during 2018 (European IVF Monitoring Consortium, 2022). Furthermore, utilization in Uruguay and Argentina is more similar to 638 cycles/million (excluding fertility preservation) reported by the CDC/USA in 2019 (https://www.cdc.gov/art/state-specific-surveillance/2019/pdf/State-Specific-ART-Surveillance-U.S.-2019-Data-Brief-h.pdf). The reason for utilization in a wealthy country like the USA being closer to LMIC in Latin America has to do with the type of reproductive policies in the majority of states in the USA and in the Americas altogether, where out-of-pocket funding prevails; this is in comparison with state funding or partial or total reimbursement in the majority of high-income countries in Europe.

The proportion of FET cycles continues to rise, representing 66.6% of all autologous transfers. This has been associated with a continuous drop in the mean number of fresh embryos transferred to 1.6.

As reported in the past, both pregnancy and delivery rates after FET were higher than after fresh transfers, irrespective of the number of embryos transferred. This might look surprising, considering that a large proportion of FET cycles result from failed fresh transfers. The main reason for this is the proportion of blastocyst transfers, which is much higher in FET (86.3%) compared with only 53.6% after fresh transfers. As seen in Table 3, Supplementary Table 2 and Figure 9, the delivery rate after elective and non-elective SET and DET were significantly higher after blastocyst transfer compared with the transfer of cleaving embryos. The beneficial role of blastocyst transfer, rather than the transfer of fresh or frozen embryos, is further examined in Figure 8, where both CPR and delivery rates were the same if only blastocysts were transferred in a group of 5867 fresh transfers and 15,169 FET. Furthermore, the transfer of embryos after a freeze-all cycle yields better pregnancy and delivery rates than after regular FET. This is because most, if not all, regular FET result from failed fresh transfers where the best embryos have already been used, while in freeze-all cases, the best blastocyst is thawed first. Again, this shows that selection of the best blastocyst for transfer is what yields the best results, either through morphology assessment or after the addition of PGT.

In 2020, for the first time, collaborating institutions were asked to describe the type of endometriosis when this was part of a primary or secondary diagnosis. This included how the diagnosis was reached, and when reached surgically (mostly laparoscopic), centres were asked to describe the type of surgery performed, classified into five categories: peritoneal fulguration, cystectomy or drainage of endometrioma, deep infiltration, partial oophorectomy and a combination of the above. Endometriosis was diagnosed by direct visualization in 11,153 out of 39,418 initiated cycles (28.3%). The number of oocytes collected as well as the delivery rate, stratified by age, were compared in 5779 cases of women having peritoneal endometriosis as the only diagnosis, excluding freeze-all cycles, compared with women having tubal and/or endocrine factors, excluding ovarian insufficiency. As seen in Supplementary Table 7, delivery rates were higher in the endometriosis group in all age categories, in spite of generating fewer oocytes. Therefore, women with a history of peritoneal endometriosis fulgurated or removed by laparoscopy seem to have better ART outcomes than women with tubal or endocrine factors. Although we understand that in the absence of a randomized trial the above statement cannot be certified, findings in this database are in agreement with a study by Opøien *et al*. (2011) who showed better ART outcomes in minimal or mild endometriosis after surgical removal of endometriotic tissue; a review by Senapati *et al.* (2016), using the SART database, agreed with the findings here, that in the absence of comorbidity, endometriosis yields fewer oocytes but higher pregnancy and delivery rates.

The number of centres and cycles reporting PGT is increasing year after year. In 2020, 76.6% of centres reported PGT, which included 24.1% of aspirations with at least one mature oocyte. PGT was used in 27,287 blastocysts, most of which were examined by next-generation sequencing. The proportion of aneuploidy was 49.8% of embryos in women ≤34 years; 59.9% of embryos in women aged 35-39, and 77.1% of embryos in women ≥40 years. Furthermore, the proportion of aneuploidy in 3166 embryos generated from oocyte donors (mean age 25.5 years) was 36.1%. As seen in Table 4, using PGT decreased miscarriage rates and increased delivery rates at all ages, including oocyte recipients. Furthermore, when comparing the outcome in oocyte recipients and autologous reproduction in women ≤34 years, miscarriage was significantly higher and delivery rates significantly lower in oocyte recipients. Nevertheless, when PGT was used, both markers improved and the differences disappeared. There is indeed a benefit in using PGT to achieve higher reproductive efficiency at all ages; however, the question is whether it is cost beneficial at all ages, which will be highly dependent on reproductive health funding policies. Irrespective of the wealth of a country, when the majority of treatments are out-of-pocket funded, most consumers belong to a subgroup of middle or high-income individuals. In this subgroup there is a triad consisting of families with fewer children, delayed childbearing and a progressive seeking for certainties. With this in mind, the question of absolute benefit of PGT prevails over the balance between costs for the intended benefit. This in part explains the increasing use of technology (PGT) to ensure, as far as possible, the birth of healthy children.

Unlike previous years, this report calculates the cumulative delivery rate from aspirations taking place only during 2020. In this cohort of 11,101 aspirations, only 4344 (39%) had surplus embryos available for future transfer. Therefore, if cumulative births are calculated starting from the whole cohort, the majority of women (61%) will not have a second chance of a birth resulting from the initial aspiration cycle. This is most likely due to the high proportion (34%) of women who were aged 40 years and older.

When the cumulative delivery rate was calculated only among women having surplus frozen embryos available for future transfers, the chance of a birth after a fresh transfer was already higher at all ages; the delta generated by the subsequent FET (cumulative) was also higher. Furthermore, the negative impact of age on reproductive efficiency is less pronounced in women generating more embryos. This is well represented by the slope of the line representing lower chances of a birth as age increases, which is less steep in women capable of generating more embryos from a single aspiration cycle (Figure 11). Another interesting finding is the better outcome after the sequential transfer of two blastocysts (1+1) compared with the simultaneous transfer of two blastocysts in women ≤34 years. Although the differences in delivery rates are not huge, the rate of multiple births is almost 20 times higher after the simultaneous transfer of two blastocysts (1.6% compared with 30.5%, respectively) than after 1+1 (Figure 12). The impact of multiple births in terms of perinatal mortality and preterm and extreme preterm births can be seen in Table 5 and Figure 13. In 2020, 65% of all multiple births resulted from women ≤34 years and oocyte recipients. Therefore, a strategy of 1+1 blastocysts in these two groups of women should significantly reduce multiple births, maintaining acceptable delivery rates.

To summarize, after more than 30 years of a south-south multinational cooperation programme among multiple institutions and countries of Latin America, we believe this to be the most efficient way of procuring regional sustainable growth. Throughout the years, numerous centres have acquired the capacity and the ability to register their data in a systematic way, which is a fundamental step towards progress. The software developed by RLA allows every centre to automatically access results of their own data and compare them with the global results of their country and sub-region. This has proved to be of immense value when developing strategies to procure a better balance between safety and efficacy, especially with the difficulties that result from a population where 34% of women are aged ≥40 years and the majority of treatments are out-of-pocket funded.

### Data availability

The data that has been used is confidential.

## APPENDIX SUPPLEMENTARY MATERIALS

**Supplementary table 1:** Data on ART were collected from 188 centres in 16 countries in Latin America

ARGENTINA
• Servicio de Medicina Reproductiva, Instituto Gamma
• Centro de Estudios en Ginecología y Reproducción (CEGYR)
• Centro Integral de Ginecología, Obstetricia y Reproducción (CIGOR)
• Centro de Medicina Reproductiva Bariloche , Fertility Patagonia
• Centro de Estudios en Reproducción y Procedimientos de Fertilización Asistida (CRECER)
• FERTILAB
• Centro de Reproducción SA
• Fertilis Medicina Reproductiva
• Fertya
• GESTAR
• Hospital de Clínicas José de San Martin
• FECUNDART
• Centro de Reproducción, servicio de Ginecología Hospital Italiano
• Mater, Medicina Reproductiva
• Nascentis, Medicina Reproductiva
• HALITUS, Instituto Médico
• PREGNA, Medicina Reproductiva
• Programa de asistencia reproductiva PROAR
• PROCREARTE
• Fertilidad San Isidro
• SARESA, Salud reproductiva Salta
• VITAE, Medicina Reproductiva
BOLIVIA
• CENALFES
• Instituto de Salud Reproductiva (ISARE)
• EMBRIOVID, centro integral de reproducción y especialidades médicas
BRAZIL
• ANDROLAB, Clínica e Laboratorio de Reproduçao Humana e Andrologia
• ANDROFERT, Centro de Referencia en Reproduçao Masculina
• FERTIVITRO, Centro de Reproduçao Humana
• BIOS, Centro de Medicina Reprodutiva
• FIV-MED
• Centro de Medicina da reproduçao
• VIDA, Centro de Fertilidade
• Clínica FERTWAY
• Nascer-Medicina Reprodutiva Ltda.
• ORIGINARE, Centro de Reproduçao Humana
• CLINIFERT, Centro de Reproduçao Humana
• CONCEPTUS, Centro de Reproduçao Asistida de Ceara
• CONCEBER, Centro de Reproduçao Humana
• Clínica Origen
• Clínica Pro-Gerar
• Centro de Reproduçao humana CONCEPTION
• Centro de Reproduçao Humana MONTELEONE
• Fértile Diagnósticos
• CEERH, Centro especializado em Reproduçao Humana
• Embrios, centro de Reproduçao humana
• EMBRYOLIFE, Instituto de Medicina Reproductiva
• CENAFERT, Centro de Medicina Reproductiva
• Instituto VERHUM
• Clínica FERTIBABY BH
• Fertilcare, Centro de Reproduçao humana Ltda.
• FECUNDA, Reproduçao Humana
• FELICCITA, Instituto de Fertilidade Ltda.
• HUMANA, Medicina Reproductiva
• FertLiv
• FERTILITY, Centro de Fertilizaçao Asistida
• FERTIL Reproduçao Humana
• REPROFERTY
• FERTICLIN, Clínica de Fertilidad Humana
• FECUNDAR Medicina Reproductiva
• Genesis Instituto de Reproduçao humana de Cascavel PR
• GENESIS, Centro de Assistencia en Reproduçao Humana
• Genics, medicina reproductiva y genómica
• FERTIPRAXIS
• GERA, Grupo de endoscopia e Reproduçao Asistida
• Nucleo de Reproduçao humana do Hospital Moinhos de Vento -GERAR
• Clinica GERAR VIDA
• Cegonha Medicina Reproductiva
• PRIMORDIA, Medicina Reproductiva
• Hospital de Clínicas de Ribeirao Preto
• HUNTINGTON Campinas
• HUNTINGTON, Centro de Medicina Reproductiva (Sao Paulo)
• JULES WHITE, Centro de Medicina Reprodutiva
• HUNTINGTON Vila Mariana
• Ideia Fertil, Santo André
• Ideia Fertil, Sao Paulo
• IMR, Instituto de Medicina Reproductiva e Fetal
• Insemine, Centro de Reproduçao Humana
• Centro de Reproduçao Humana Santa Joana
• Life Reproduçao humana
• FERTILITAT, Centro de Medicina Reproductiva
• Clinica Nidus
• Centro de Pesquisa e Reproduçao Humana Nilo Frantz
• Origen, Centro de Medidicina Reproductiva BH
• Procriar, Centro de Medicina Reproductiva e diagnósticos Ltda., Blumenau
• Clinica PRO-CRIAR, Medicina Reproductiva BH
• Clinica PRO NASCER
• Clinica ProSer
• Centro de Reproduçao Humana de Sao Jose de Rio Preto
• Centro de fertilidad Hospital Moinhos de vento
• GENESIS, Centro de Reproduçao Humana
• Centro de Reproduçao Humana Prof. Franco Junior
• Centro de Ensino e Pesquisa em Reproduçao Asistida (CEPRA)
CHILE
• UMR Clínica de la Mujer Antofagasta
• Centro de Estudios Reproductivos (CER)
• Unidad de Medicina Reproductiva, Clínica Alemana
• Unidad de Medicina Reproductiva, Clínica las Condes
• Unidad de Medicina Reproductiva, Clínica de la Mujer
• UMR clínica Indisa
• Programa de Fertilización Asistida I.D.I.M.I.
• Clínica Monteblanco
• Instituto de Medicina Reproductiva Concepción S.A.
• Centro de reproducción humana, Valparaíso
• SG Fertility Chile
COLOMBIA
• Centro FECUNDAR, Cali
• Unidad de fertilidad del Coutry ltda. CONCEPTUM
• Asociados en Fertilidad y Reproducción Humana
• Centro de fertilidad Clínica de la mujer
• Clínica Eugin
• FERTIVIDA
• Clínica Machicado SAS
• Centro Médico IMBANACO
• Instituto de Fertilidad Humana S.A.S. (INSER Bogotá)
• IN SER, Instituto Antioqueño de Reproducción (Medellín)
• Procrear
• Profamilia Fertilidad
• Unidad de Fertilidad, Procreación Medicamente Asistida
• Unión temporal IN SER eje cafetero (Pereira)
COSTA RICA
• Azul Fertility expert
ECUADOR
• Clínica INFES
• Instituto Nacional de Investigación de Fertilidad y Esterilidad (INNAIFEST)
• CONCEBIR, Unidad de Fertilidad y Esterilidad
• Centro Ecuatoriano de Reproducción Humana
GUATEMALA
• Centro de Reproducción Humana S.A. (CER)
• Centro Clínico Gestar (nuevo)
MEXICO
• Biofertility Center
• Centro de Diagnóstico Ginecológico
• Clínica Cerh S e RL de CV
• Dr. Cigüeña
• URA, Unidad de reproducción asistida de Hospital CIMA Hermosillo
• Centro de Cirugía Reproductiva y Ginecología, Unidad de Fertilización In Vitro (REPROGYN)
• Instituto de Innovación Tecnológica y Medicina Reproductiva CITMER (Ciudad de México)
• Centro de Innovación tecnológica y medicina Reproductiva (Monterrey)
• Citmer-Centro de innovación tecnológica y medicina reproductiva Puebla
• Instituto para el estudio de la Concepción Humana IECH
• Centro de Reproducción Asistida del Hospital Español (HISPAREP)
• Centro de Reproducción Asistida del Occidente
• Centro de Reproducción Asistida de Saltillo
• Centro Universitario de Medicina Reproductiva
• Fertility Center Cancún
• Fertilita Medicina Reproductiva, Laboratorio in vitro
• Fertygen
• Centro de Medicina reproductiva Filius
• Genesis Centro de Fertilidad (Culiacán)
• Ginecología y Reproducción Asistida GYRA
• Unidad de Medicina Reproductiva del Hospital Ángeles del Pedregal
• IECH de Baja California
• Instituto Mexicano de Alta Tecnología Reproductiva S.C. (INMATER)
• Concibo
• Instituto Médico de la mujer (RED CREA)
• Instituto VIDA Guadalajara-Instituto de Ciencias en Reproducción Humana
• Instituto de Ciencias en Reproducción Humana, VIDA sede Matamoros
• Centro especializado para la atención de la mujer (CEPAM)
• INGENES DF
• INGENES Guadalajara
• Ingenes Monterrey
• Instituto de Ciencias en Reproducción Humana (VIDA), sede León
• MasFertil
• Instituto de ciencias en reproducción humana del Sureste (Vida Mérida)
• Clínica Nascere
• Plenus, Reproducción Asistida
• PROGEN
• Clínica de Infertilidad y reproducción asistida de Toluca SA de CV
• Instituto de Ciencias en reproducción humana VIDA, ciudad de México.
• Centro CARE
• Vida, Instituto de Reproducción Humana del Noroeste, Tijuana
NICARAGUA
• Centro de Fertilidad de Nicaragua
PANAMA
• IVI Panamá S.A.
• Instituto de salud femenina
• IVF Panamá Centro de reproducción Punta Pacífica (PTY)
PARAGUAY
• Neolife, Medicina y cirugía reproductiva
PERU
• Clínica CEFRA, Centro de Fertilidad y Reproducción Asistida
• CEFERGIN
• Centro de Fertilidad y Ginecología del Sur (CFGS)
• Clínica de fertilidad del norte, Clinifer de Chiclayo
• FERTILAB
• Centro de Fertilidad Germinar
• Inmater, Clinica de fertilidad y reproducción asistida
• Instituto de Reproducción de la Clínica Ricardo Palma
• Clínica Miraflores, Instituto de Ginecología y Fertilidad
• Nacer, centro de reproducción humana de Lima
• NiuVida
• Grupo Pranor San Isidro, Clínica CONCEBIR
• Grupo Pranor, Instituto de Ginecología y Reproducción Monterrico
REPUBLICA DOMINICANA
• Instituto de reproducción y ginecología del Cibao - IREGCI
• Programa de fertilización asistida y medicina perinatal - PROFERT
URUGUAY
• Centro de Esterilidad Montevideo (CEM)
• Centro de Reproducción Humana del Interior
VENEZUELA
• FERTILAB

**Supplementary Table 2:** Clinical pregnancy rate, delivery rate and gestational order in elective and non-elective blastocyst SET and DET in Fresh autologous IVF/ICSI in 2020.

Type of transfer	Embryo transfers		Clinical pregnancies		Deliveries							
	**Number**	**%**	**Number**	**%**	**Number of deliveries**	**Delivery rate per embryo transfer**	**Singleton (n)**	**Singleton (%)**	**Twin** **(n)**	**Twin** **(%)**	**≥Triplets (n)**	**≥Triplets (%)**
**eSET**	1,466	57.1	654	44.6	501	34.2	496	99.0	5	1.0	0	0
**eSET**	1,466	57.1	654	44.6	501	34.2	496	99.0	5	1.0	0	0
**oDET**	1,458	48.3	513	35.2	340	23.3	276	81.2	62	18.2	2	0.6
**eDET**	1,560	51.7	850	54.5	591	37.9	411	69.5	180	30.5	0	0

eSET, elective single-embryo transfers; oSET, transfer of only one embryo because there are no more embryos available for transfer; eDET, elective double-embryo transfers; oDET, the transfer of only two embryos because there are no more embryos available for transfer.

**Supplementary Table 3:** Clinical pregnancy rate, delivery rate and gestational order according to the number of embryos transferred in Fresh OD in 2020.

Number of embryos transferred	Embryo transfers		Clinical pregnancies		Deliveries							
	Number	%	Number	CPR (%)	Number of deliveries	Delivery rate per embryo transfer (%)	Singleton (n)	Singleton (%)	Twin (n)	Twin (%)	≥Triplets (n)	≥Triplets (%)
**1**	1,157	37.4	511	44.2	373	32.2	367	98.4	6	1.6	0	0
**2**	1,622	52.5	789	48.6	582	35.9	414	71.1	167	28.7	1	0.2
**≥3**	312	10.1	164	52.6	127	40.7	69	54.3	56	44.1	2	1.6
**Total**	3,091	100	1,464	47.4	1,082	35.0	850	78.6	229	21.2	3	0.2

**Supplementary Table 4:** Clinical pregnancy rate, delivery rate and gestational order according to the number of embryos transferred in Vitrified/warmed OD in 2020.

Number of embryos transferred	Embryo transfers		Clinical pregnancies		Deliveries							
	Number	%	Number	CPR (%)	Number of deliveries	Delivery rate per embryo transfer (%)	Singleton (n)	Singleton (%)	Twin (n)	Twin (%)	≥Triplets (n)	≥Triplets (%)
**1**	3,637	56.0	1,482	40.8	1,085	29.8	1,071	98.7	14	1.3	0	0
**2**	2,376	36.6	1,109	46.7	750	31.6	569	75.9	177	23.6	4	0.5
**≥3**	477	7.4	252	52.8	159	33.3	94	59.1	58	36.5	7	4.4
**Total**	6,490	100	2,843	43.8	1,994	30.7	1,734	87.0	249	12.5	11	0.5

**Supplementary Table 5:** Clinical pregnancy rate, delivery rate and gestational order according to the number of embryos transferred in Autologous FET in 2020.

Number of embryos transferred	Embryo transfers		Clinical pregnancies		Deliveries							
	Number	%	Number	CPR (%)	Number of deliveries	Delivery rate per embryo transfer (%)	Singleton (n)	Singleton (%)	Twin (n)	Twin (%)	≥Triplets (n)	≥Triplets (%)
**1**	11,994	54.1	4,571	38.1	3,303	27.5	3,253	98.5	50	1.5	0	0
**2**	9,319	42.0	4,251	45.6	2,885	31.0	2,227	77.2	647	22.4	11	0.4
**≥3**	865	3.9	357	41.3	235	27.2	176	74.9	57	24.3	2	0.8
**Total**	22,178	100	9,179	41.4	6,423	29.0	5,656	88.1	754	11.7	13	0.2

This data includes all embryo transfers from autologous FET cycles, including cases of FET from previously failed fresh transfers; FET from Freeze all cycles, FET from PGT cycles and FET from FTO cycles.

**Supplementary Table 6:** Clinical pregnancy rate, delivery rate and gestational order according to the number of embryos transferred after Autologous Freeze all cycles, 2020.

Number of embryos transferred	Embryo transfers		Clinical pregnancies		Deliveries							
	Number	%	Number	CPR (%)	Number of deliveries	Delivery rate per embryo transfer (%)	Singleton (n)	Singleton (%)	Twin (n)	Twin (%)	≥Triplets (n)	≥Triplets (%)
**1**	3501	46.8	1246	35.6	835	23.9	816	97.7	19	2.3	0	0
**2**	3554	47.5	1713	48.2	1131	31.8	860	76.0	262	23.2	9	0.8
**≥3**	429	5.7	195	45.5	126	29.4	89	70.6	35	27.8	2	1.6
**Total**	7484	100	3154	42.1	2092	28.0	1765	84.4	316	15.1	11	0.5

This data includes all embryo transfers performed in 2020 from autologous Freeze all FET cycles. Cases with PGT are not included.

**Supplementary Table 7:** Effect of peritoneal endometriosis in ART outcome, 2020.

Age (years)	Diagnosis	n	Oocytes retrieved	Mean number	Deliveries	Transfers	Delivery rate per transfer
<35	Endometriosis peritoneal	1501	13,943	9.3^*^	459	1199	38.3
	Tubal and other endocrine factors^*^	679	7888	11.6^*^	191	563	33.9
35 - 39	Endometriosis peritoneal	2322	16,468	8.9	527	1688	31.2^*^
	Tubal and other endocrine factors^*^	989	8739	8.8	179	743	24.1^*^
>39	Endometriosis peritoneal	1956	10,196	5.2^*^	171	1018	16.8^*^
	Tubal and other endocrine factors^*^	771	4649	6.0^*^	46	377	12.2^*^

n: number of oocyte retrievals with a history of peritoneal endometriosis and tubal and endocrine factors, excluding freeze all cases. Tubal and endocrine factors exclude endometriosis as second diagnosis. (^*^) Significantly different

Mean number of oocytes:

**<35:** 9.3±6.274 *versus* 11.6±7.201: *p*<0.0001 (95% CI 2.1171 to 2.4829)

**35-39:** 8.9±5.368 *versus* 8.8±6.084: *p*=0.1793 (95% CI -0.2460 to 0.0460)

**>39:** 5.2±4.415 *versus* 6.0±5.327: *p*<0.0001 (95% CI 0.6363 to 0.9637)

Delivery rate/Transfer:

**<35:** 38.3 *versus* 33.9: *p*=0.0744 (95% CI -0.4378 to 9.1025)

**35-39:** 31.2 *versus* 24: *p*=0.0004 (95% CI 3.2333 to 10.8003)

**>39:** 16.8 *versus* 12.2: *p*=0.0353 (95% CI 0.3185 to 8.3988)

## References

[r1] Dyer SJ, Chambers G, Zegers-Hochschild F, Adamson GD. (2019). Access to ART: concepts indicators, impact. Hum Reprod.

[r2] Wyns C, De Geyter C, Calhaz-Jorge C, Kupka MS, Motrenko T, Smeenk J, Bergh C, Tandler-Schneider A, Rugescu IA, Goossens V, European IVF Monitoring Consortium (EIM), for the European Society
of Human Reproduction and Embryology (ESHRE); (2022). ART in Europe, 2018: results generated from European registries
by ESHRE. Hum Reprod Open.

[r3] Grant RL. (2014). Converting an odds ratio to a range of plausible relative risks
for better communication of research findings. BMJ.

[r4] Opøien HK, Fedorcsak P, Byholm T, Tanbo T. (2011). Complete surgical removal of minimal and mild endometriosis
improves outcome of subsequent IVF/ICSI treatment. Reprod Biomed Online.

[r5] Practice Committee of the Society for Reproductive Endocrinology and
Infertility, Quality Assurance Committee of the Society for Assisted
Reproductive Technology, and the Practice Committee of the American
Society for Reproductive Medicine (2022). Multiple gestation associated with infertility therapy: a
committee opinion. Fertil Steril.

[r6] Sazonova A, Källen K, Thurin-Kjellberg A, Wennerholm UB, Bergh C. (2013). Neonatal and maternal outcomes comparing women undergoing two in
vitro fertilization (IVF) singleton pregnancies and women undergoing one IVF
twin pregnancy. Fertil Steril.

[r7] Senapati S, Sammel MD, Morse C, Barnhart KT. (2016). Impact of endometriosis on in vitro fertilization outcomes: an
evaluation of the Society for Assisted Reproductive Technologies
Database. Fertil Steril.

[r8] Zegers-Hochschild F, Adamson GD, Dyer S, Racowsky C, de Mouzon J, Sokol R, Rienzi L, Sunde A, Schmidt L, Cooke ID, Simpson JL, van der Poel S. (2017). The International Glossary on Infertility and Fertility Care,
2017. Hum Reprod.

[r9] Zegers-Hochschild F, Crosby JA, Musri C, Souza MDCB, Martinez AG, Silva AA, Mojarra JM, Masoli D, Posada N. (2020). Assisted reproductive techniques in Latin America: The Latin
American Registry, 2017. JBRA Assist Reprod.

[r10] Zegers-Hochschild F, Crosby JA, Musri C, Souza MDCB, Martínez AG, Silva AA, Mojarra JM, Masoli D, Posada N. (2021). Celebrating 30 years of ART in Latin America; and the 2018
report. JBRA Assist Reprod.

[r11] Zegers-Hochschild F, Crosby JA, Musri C, Souza MDCB, Martinez AG, Silva AA, Mojarra JM, Masoli D, Posada N, Reproduction LANOA (2022). Assisted reproductive technologies in Latin America: the Latin
American Registry, 2019. JBRA Assist Reprod.

