# Corrigendum to “ART in Latin America: The Latin American Registry, 2020” [JBRA Assist Reprod. 2023;27(3):514-538]

**DOI:** 10.5935/1518-0557.20240064

**Published:** 2024

**Authors:** Fernando Zegers Hochschild, Javier A. Crosby, Carolina Musri, Fanny Petermann-Rocha, Maria do Carmo Borges de Souza, A. Gustavo Martinez, Ricardo Azambuja, Armando Roque, Gustavo Estofan, Mario Vega Croker, on behalf of the Latin American Network of Assisted Reproduction

**Affiliations:** 1 Unit of Reproductive Medicine, Clínica Las Condes, Santiago, Chile; 2 Program of Ethics and Public Policies in Human Reproduction, Facultad de Medicina, Universidad Diego Portales, Santiago, Chile; 3 Centro de Investigación Biomédica, Facultad de Medicina, Universidad Diego Portales, Santiago, Chile; 4 Fertipraxis, Centro de Reproducao Humana, Rio de Janeiro, Brazil; 5 Medicina Reproductiva Fertilis, San Isidro, Buenos Aires, Argentina; Universidad de Belgrano, Buenos Aires, Argentina; 6 Fertilitat, Centro de Medicina Reprodutiva, Porto Alegre, RS90620-130, Brazil; 7 Centro Especializado de Atención a la Mujer (CEPAM), Hacienda de las Palmas, Huixquilucan, Estado de México, Mexico; 8 CIGOR, Córdoba, Argentina; 9 Panamá Fertility, Consultorios Hospital Punta Pacífica, Ciudad de Panamá, Panama; 10 Latin American Network of Assisted Reproduction (REDLARA), Montevideo, Uruguay

The authors regret that in Supplementary Table 7 (Effect of peritoneal endometriosis in ART outcome) several cases were wrongly allocated as peritoneal endometriosis. After correcting the data to include all cases where the primary or secondary diagnosis was endometriosis, as diagnosed by direct observation (laparoscopy), re-analysis revealed that the differences in delivery rate were not significant. Correct supplementary table and revised results and discussions have been provided below in order to reflect the new analysis:

**Supplementary Table 7:** Effect of peritoneal endometriosis in ART outcome, 2020.

Age (years)	Diagnosis	N	Oocytes retrieved	Mean number	Deliveries	Transfers	Delivery rate per transfer
<35	Endometriosis	200	1875	9.51±5.57	53	142	37.3%
	Tubal and other endocrine factors	851	8935	10.50±6.86^*^	222	630	35.2%
35 - 39	Endometriosis	381	2618	6.87±5.49	66	225	29.3%
	Tubal and other endocrine factors	1181	9813	10.28±6.87^*^	207	861	24.0%
>39	Endometriosis	172	962	5.59±5.11	10	66	15.2%
	Tubal and other endocrine factors	807	4946	6.12±5.44^*^	55	437	12.6%

n: number of oocyte retrievals with a history of endometriosis and tubal and endocrine factors, excluding freeze all cases. Tubal and endocrine factors exclude endometriosis as second diagnosis. (^*^) Significantly different.

## REVISED RESULTS TEXT

Endometriosis, as diagnosed by direct observation (laparoscopy), was present, either as a primary or secondary diagnosis, in 753 out of 39,418 initiated fresh cycles (1.9%). Laparoscopic observation included 67% of peritoneal fulguration, 22% of surgery for ovarian cysts, deep infiltration, or a combination of both, and 11% cases where no specific description was registered. A comparison was made between the outcome of cases where endometriosis was diagnosed, and a ‘control group’ (n=2839) of tubal and endocrine factors excluding premature ovarian insufficiency (Supplementary Table 7). In this ‘control group’, cases with a secondary diagnosis of endometriosis were also ruled out. Supplementary Table 7 provides information on the numbers and the mean number of oocytes collected, as well as the delivery rates in these two groups of women, stratified by age categories. Although the mean number of oocytes collected at all ages was significantly lower in the presence of endometriosis (<35: 9.51 [5.57] *versus* 10.50 [6.86]: *p*<0.0001 (95% CI 0.6661 to 1.3139); 35-39: 6.87 [5.49] *versus* 10.28 [6.87]: *p*<0.0001 (95% CI 3.1253 to 3.6947); >39: 5.59 [5.11] *versus* 6.12 [5.44]: *p*=0.0053 (95% CI 0.1578 to 0.9022), the delivery rate per embryo transfer was not significantly different between the endometriosis and control groups (<35: 37.3% versus 35.2%: *p*=0.6370 (95% CI -6.3307% to 11.0585%); 35-39: 29.3% *versus* 24.0%: *p*=0.1027 (95% CI -1.0005% to 12.1257%); >39: 15.2% *versus* 12.6%: *p*=0.5579 (95% CI -4.9503% to 13.5113%).

## REVISED DISCUSSION TEXT

In 2020, for the first time, collaborating institutions were asked to describe the type of endometriosis when this was part of a primary or secondary diagnosis. This included how the diagnosis was reached, and when reached surgically (mostly laparoscopic), centers were asked to describe the type of surgery performed, classified into five categories: peritoneal fulguration, cystectomy, or drainage of endometrioma, deep infiltration, partial oophorectomy and a combination of the above. Endometriosis was diagnosed by direct visualization in 753 out of 39,418 initiated cycles (1.9%). The number of oocytes collected as well as the delivery rate, stratified by age, were compared in women having endometriosis either as a primary or secondary diagnosis, excluding freeze-all cycles, and women having tubal and/or endocrine factors, excluding ovarian insufficiency. As seen in Supplementary Table 7, in spite of the fact that the number of eggs recovered in cases with endometriosis was significantly lower at all ages the delivery rates appeared to be higher in women with endometriosis compared with women with tubal or endocrine factors, although the difference was not statistically significant. These findings follow a similar direction of a study by Opøien et al. (2011) who showed better ART outcomes in minimal or mild endometriosis after surgical removal of endometriotic tissue; and a review by Senapati et al. (2016), using the SART database, showing, that in the absence of comorbidity, endometriosis yields fewer oocytes but higher pregnancy and delivery rates.

The authors would like to apologize for any inconvenience caused.

